# Simplified computed tomography pulmonary angiography score predicts clinical deterioration in patients with acute pulmonary embolism

**DOI:** 10.1016/j.ijcha.2025.101712

**Published:** 2025-06-03

**Authors:** Jian-Kuan Yeh, Po-Wei Chen, Wei-Ting Chang, Pin-Hsuan Chiu, Pei-Fang Su, Chih-Hsin Hsu, Chih-Chan Lin, Hsien-Yuan Chang

**Affiliations:** a Division of Cardiology, department of Internal Medicine, National Cheng Kung University Hospital, College of Medicine, National Cheng Kung University, Tainan, Taiwan; b Institute of Clinical Medicine, College of Medicine, National Cheng Kung University, Tainan, Taiwan; c School of Medicine and Doctoral Program of Clinical and Experimental Medicine, College of Medicine and Center of Excellence for Metabolic Associated Fatty Liver Disease, National Sun Yat-sen University, Kaohsiung, Taiwan; d Division of Cardiology, Department of Internal Medicine, Chi Mei Medical Center, Tainan, Taiwan; e The Center for Quantitative Sciences, Clinical Medicine Research Center, National Cheng Kung University Hospital, Tainan, Taiwan; f Department of Statistics, College of Management, National Cheng Kung University, Tainan, Taiwan; g Division of Cardiology, Department of Internal Medicine, An Nan Hospital, China Medical University, Tainan, Taiwan

**Keywords:** Pulmonary embolism, CTPA, Clinical deterioration

## Abstract

**Background:**

Currently, simplified methods based on computed tomography pulmonary angiography (CTPA) to predict clinical deterioration in patients with acute pulmonary embolism (PE) are lacking. We developed a simplified imaging model with good clinical accessibility to predict this outcome.

**Study design and methods:**

Patients with acute pulmonary embolism from 2008 to 2019 were retrospectively enrolled from two medical centers to form a study cohort and a validation cohort. Seven models of pulmonary artery obstruction index (PAOI) were developed based on the location and degree of obstruction. The outcome of interest was clinical deterioration during hospitalization. Logistic regression analysis was used to assess the association between different models and clinical deterioration. The category-free net reclassification improvement (NRI) and integrated discrimination improvement (IDI) were used to quantify improvements in predictability.

**Results:**

The study group included 210 patients (mean age: 65 ± 16 years; male: 40 %) and the external validation group included 109 patients (mean age: 64 ± 17 years; male: 43 %). Calculating the nearly total obstruction of 20 peripheral arteries demonstrated good predictive ability (AUC: 0.77). Total obstruction of six peripheral arteries did not increase the odds of clinical deterioration, while total obstruction of ten peripheral arteries nearly doubled the risk of deterioration. Combining PAOI with the simplified pulmonary embolism severity index (sPESI) improved the predictive ability for clinical deterioration compared to using sPESI alone (NRI: 0.09–0.12; IDI: 0.05–0.09).

**Conclusion:**

Calculating totally obstructed pulmonary arteries simplifies the prediction of clinical deterioration. The combination of PAOI and sPESI enhances the ability to predict clinical deterioration in patients with acute PE.

## Graphical abstract

**Figure:**
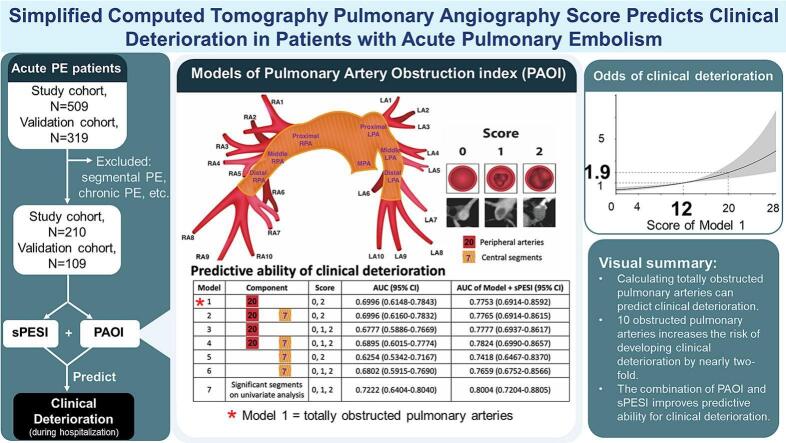

## 1 Introduction

Acute pulmonary embolism (PE), following myocardial infarction and stroke, is the third most common cardiovascular disease [1]. The annual incidence of PE is approximately 1 per 1000 people [2]. PE occurs when emboli occlude the pulmonary arteries and may cause complications, such as arrhythmias, right ventricular (RV) failure, cardiogenic shock, and even death [3]. Effective risk stratification is crucial for treating PE, as it helps identify patients that may benefit from thrombolytic therapies on top of systemic anticoagulation. The 2019 European Society of Cardiology (ESC) guideline on PE classifies patients into different risk groups based on the presence of hemodynamic instability, RV dysfunction, elevated cardiac enzymes, and the PE severity index (PESI) [4]. Thrombolytic therapy is only recommended for high-risk patients with PE, who are defined as having hemodynamic instability. For intermediate-risk patients, while thrombolytic therapy may improve outcomes, it also carries an increased risk of bleeding [5]. However, advancements in catheter-directed thrombolysis have reduced this bleeding risk, prompting a reevaluation of the benefits of early thrombolysis in intermediate-risk patients without hemodynamic instability. A key challenge, however, is that acute RV failure may develop several days after the initial embolic event, leading to hemodynamic instability and clinical deterioration. Additionally, only 5 % of intermediate-risk patients will experience such deterioration [5], highlighting the importance of promptly identifying those at risk. Despite this need, clinical predictors for identifying patients prone to clinical worsening are currently lacking, underscoring the need for more accurate prediction methods to guide treatment decisions.

Miller et al. first developed an index based on invasive pulmonary angiography to describe the thrombus burden and the severity of PE to evaluate the treatment response of systemic thrombolysis. However, the clinical utility of the Miller index is limited by its invasive nature [6]. Qanadli et al. later developed an index, the computed tomography obstruction index (CTOI) based on computed tomography pulmonary angiography (CTPA) which correlated well with the Miller index and could predict RV dilatation. The CTOI is of limited clinical utility due to its complexity in score calculation [7]. Studies revealed that the CTOI is strongly predictive of high-risk patients [8,9]. However, other studies revealed no significant correlation between the thrombus burden and clinical risks [10,11]. Based on a recently-published study, the reading time for urgent or stat priority CTPA by radiologists is between 19.6 and 31.6 h [12]. If the CTOI is calculated for risk assessment, it may take even more time and may be challenging for physicians in an emergent setting.

This study primarily aimed to develop a simplified imaging model with good clinical accessibility to predict the clinical deterioration of patients with acute PE.

## 2 Methods

### 2.1 Materials

This retrospective study used the cardiovascular databank of the National Cheng Kung University Hospital. The enrollment period spanned from January 1, 2008, to December 31, 2019, and included patients diagnosed with PE under the International Classification of Disease, ninth or tenth revision. This study adhered to the Declaration of Helsinki and obtained approval from the Human Research and Ethics Committee of the National Cheng Kung University Hospital (IRB number: B-ER-109-102). An additional group of patients from Chi Mei Medical Center, which is another tertiary medical center, from January 1, 2008, to December 31, 2021, was included for external validation. The external validation group also received approval from the Human Research and Ethics Committee of the Chi Mei Medical Center (IRB number: CMMC11011-002).

This study applied certain exclusion criteria. Patients with septic, tumor, or fat emboli, tumor invasion or pulmonary artery encasement, and stump thrombosis were excluded from the study, as well as patients with only segmental or subsegmental PE, where the thrombi were deemed too small to cause clinical symptoms and that it may be difficult to discern vessel wall and small thrombi in earlier CT images. Further, cases with poor image quality or no diagnostic CTPA were excluded. Furthermore, patients with CT-defined chronic thrombus were excluded due to differing prognoses. The simplified PESI (sPESI) was calculated [13]. The administration of systemic or catheter-directed thrombolysis was recorded for each patient.

### 2.2 Obstruction index determination

Two specialists, who were unaware of the patient’s clinical data, evaluated the images independently. Two indices, namely the Miller obstruction index and the CTOI, were calculated based on CTPA. The Miller obstruction index is calculated as Σ (*n.d*), where n represents the number of segmental arteries with thrombus (range: 1–16) and d is the presence (1) or absence (0) of obstruction. According to Miller et al., the right pulmonary artery has nine major segmental arteries (three to the upper lobe, two to the middle lobe, and four to the lower lobe), and the left pulmonary artery has seven major segmental arteries (two to the upper lobe, two to the lingula, and four to the lower lobe) [6]. The CTOI is calculated as Σ (*n.d*)/40 × 100, where n is the number of segmental arteries with thrombus (ranging from 1 to 20), and d is the degree of obstruction (range: 0–2). Both pulmonary arteries have ten segmental arteries (three to the upper lobes, two to the middle lobe and lingula, and five to the lower lobes). The degree of obstruction ranges from 0 (no obstruction) to 1 (partial obstruction) to 2 (nearly total obstruction) [7].

**Fig. 1:**
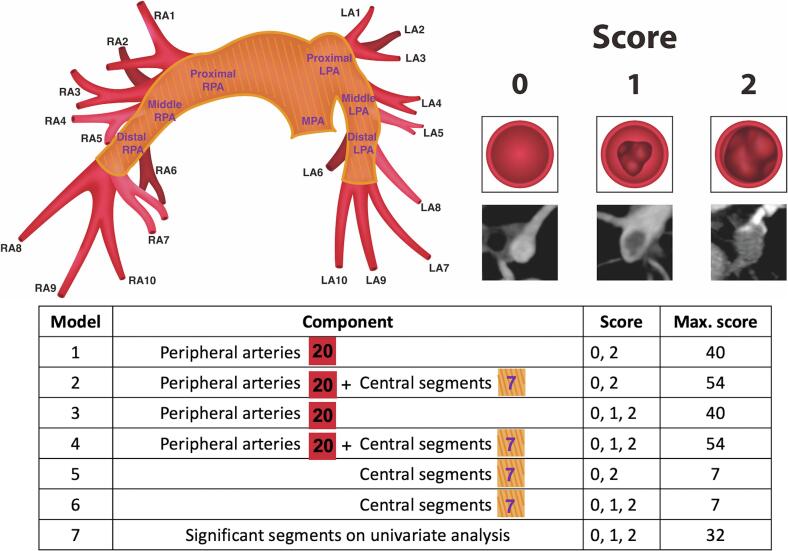
Flow Diagram of the Study Cohort. Abbreviations: CT, computed tomography; CTA, computed tomography angiography; PE, pulmonary embolism.

The degree of obstruction of the central segments was also scored, using the same scale of 0 (no obstruction) to 1 (partial obstruction) to 2 (nearly total obstruction), to comprehensively assess the pulmonary circulation and evaluate the entire pulmonary vasculature. The central pulmonary artery was divided into seven segments based on the lung lobes they supply: the main pulmonary artery, the proximal, middle, and distal parts of bilateral pulmonary arteries. The proximal segment of the right and left pulmonary arteries represents the area between the branching from the main pulmonary artery to the branching of the segmental arteries to the upper lobe. The middle segment lies between the upper lobe segmental arteries and the middle lobe segmental arteries, while the distal segment is distal to the segmental arteries to the middle lobe. The transverse section of the pulmonary artery by direct visual estimation primarily determines the obstruction percentage (Fig. 1). When inter-reader disagreements were present, the two specialists read the CTPA together, and the final scoring was determined.

### 2.3 Proposed models

Six models were developed based on the location (central versus peripheral) and the degree (nearly total versus partial) of obstruction. Partially-occluded pulmonary arteries were included for a semi-quantitative estimation of the total plaque burden. The peripheral arteries are the segmental pulmonary arteries. Model 1 represents the sum of nearly totally obstructed peripheral arteries. Model 2 is the sum of nearly totally obstructed central and peripheral arteries. Model 3 contains the CTOI, which is the sum of peripheral pulmonary artery obstruction. Model 4 combines central and peripheral pulmonary artery obstruction. Model 5 comprises the sum of nearly totally obstructed central arteries. Model 6 is the sum of central pulmonary artery obstruction. A seventh model, in addition to the six pre-specified models, was developed by performing univariate logistic regression and selecting the statistically significant parts of pulmonary arteries that impact the outcome (Fig. 1). The analysis included the combination of models 1 to 7 together with the sPESI. Further discussions were conducted when there is a discrepancy in judgment until a conclusion is reached.

### 2.4 Outcomes

Two specialists carefully reviewed the electric medical records of enrolled patients. The outcome included clinical deterioration, which is death from PE, cardiopulmonary resuscitation, mechanical ventilation, vasopressor therapy for systemic arterial hypotension, thrombolysis, catheter-directed therapy, and surgical embolectomy [14]. Each inpatient was followed up until discharged, while outpatients were followed up for 1 month. PE-related death was analyzed as a separate outcome.

### 2.5 Statistical analysis

Continuous data are presented as the mean ± standard deviation while dichotomous data as numbers and percentages. The Wilcoxon Rank Sum test was used for comparisons of continuous variables. Fisher’s exact test was used for categorical variables.

Variables associated with clinical deterioration were identified using univariate logistic regression analysis along with Bonferroni correction and false discovery rate. Multivariate logistic regression was used to analyze the combination of different models plus sPESI. Model performance was calculated by the area under the curve (AUC) of the receiver operating characteristic curve. The accuracy, sensitivity, and specificity were calculated. The category-free NRI and IDI were used to quantify the improvement of the proposed models plus sPESI in comparison with the reference model, which is the sPESI in our study. The Bland-Altman analysis of agreement and the interanalysis correlation coefficient were used to assess the intra- and inter-rater reliability. Finally, a nomogram combining the imaging model and sPESI was proposed. Statistical software R (Version 4.0.2 for Windows) was used for all statistical tests. All statistical tests were 2-sided, and a *P*-value of <0.05 was considered statistically significant.

## 3 Results

### 3.1 Study population

**Fig. 2:**
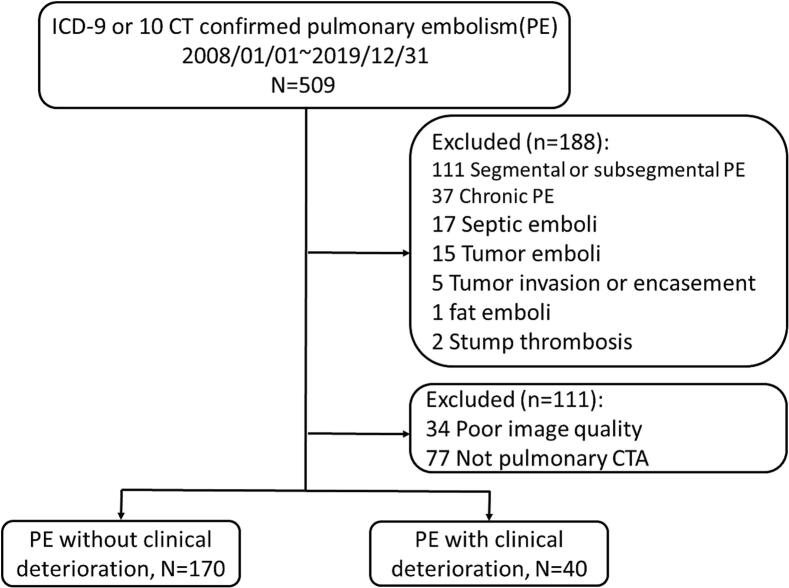
Illustration of Models based on Central and Peripheral Pulmonary Arteries and Degree of Obstruction. The left upward illustration is a schematic representation of the pulmonary arterial vasculature. The area in red represents the 20 peripheral segmental pulmonary arteries and the area shaded in yellow represents the central pulmonary arteries, which is further divided into the proximal, middle, and distal parts based on the lung lobes which they supply. The right upward illustration is the scores based on levels of obstruction. The illustrations and the reference on computed tomography are listed below. A score of 0 indicates no thrombus, a score of 1 indicates partial obstruction, and a score of 2 indicates nearly total obstruction. The below table is an explanation of models 1 to 7. Model 1 represents the sum of nearly total obstructed peripheral arteries. Model 2 is the sum of nearly total obstructed central and peripheral arteries. Model 3 includes the sum of peripheral pulmonary artery obstruction, which is the computed tomography obstruction index. Model 4 combines central and peripheral pulmonary artery obstruction. Model 5 comprises the sum of nearly total obstructed central arteries. Model 6 is the sum of central pulmonary artery obstruction. Abbreviations: LA1-10, 1st to 10th segmental pulmonary arteries of the left pulmonary artery; LPA, left pulmonary artery; RA1-10, 1st to 10th segmental pulmonary arteries of the right pulmonary artery; RPA, right pulmonary artery.

A total of 509 patients with PE were retrospectively screened. The exclusions comprised 111, 37, 40, and 111 patients with only segmental or subsegmental PE, chronic PE, other PE etiologies, and suboptimal image quality for evaluation, respectively (Fig. 2). Finally, the analysis included 210 patients (age: 65 ± 16 years; male: 40 %), including 170 (81 %) with no clinical deterioration and 40 (19 %) with clinical deterioration. The baseline clinical characteristics were balanced between patients with and without clinical deterioration apart from the sPESI. The sPESI was statistically significantly higher in the group with clinical deterioration (2.55 ± 1.47 vs. 1.42 ± 1.13, *P* < 0.01). Among the 40 patients with clinical deterioration, 21 (53 %) received systemic thrombolysis. The Miller index and the CTOI were higher in the group with clinical deterioration (Table 1).

**Table 1:** Baseline Characteristics of Patients with Acute Pulmonary Embolism with and without Clinical Deterioration.

	Overall (N = 210)	With clinical deterioration (N = 40)	Without clinical deterioration (N = 170)	p-value
Age (years old)	64.98 ± 16.18	65.47 ± 17.44	64.86 ± 15.92	0.66
Sex (male)	85 (40 %)	18 (45 %)	67 (39 %)	0.59
Body mass index (kg/m^2^)	25.99 ± 5.84	25.26 ± 4.93	26.16 ± 6.03	0.98
sPESI	1.63 ± 1.28	2.55 ± 1.47	1.42 ± 1.13	0<.01
Cancer	65 (31 %)	12 (30 %)	53 (31 %)	0.99
Hypertension	115 (55 %)	23 (58 %)	92 (54 %)	0.73
Diabetes mellitus	43 (20 %)	6 (15 %)	37 (22 %)	0.39
Coronary artery disease	19 (9 %)	3 (8 %)	16 (9 %)	0.99
Heart failure	27 (13 %)	6 (15 %)	21 (13 %)	0.61
Atrial fibrillation	26 (12 %)	7 (18 %)	19 (11 %)	0.29
Old stroke	19 (9 %)	3 (8 %)	16 (9 %)	0.99
Risk				
High	15 (7 %)	11 (28 %)	4 (2 %)	0<.01
Intermediate	119 (57 %)	23 (58 %)	96 (56 %)	
Low	76 (35 %)	6 (15 %)	70 (41 %)	
Systemic t-PA	21 (10 %)	21 (53 %)	0 (0 %)	0<.01
Miller index	11.30 ± 4.33	12.88 ± 3.28	10.93 ± 4.47	0.01
CTOI	20.37 ± 9.31	25.27 ± 7.90	19.21 ± 9.26	0<.01

Data are presented as mean ± SD or N (%).

Abbreviations: sPESI, simplified Pulmonary Embolism Severity Index.

This study retrospectively screened 319 patients from another tertiary medical center, with 210 patients being excluded (Fig. S1). Finally, the external validation included 109 patients (age: 64 ± 17 years; male: 43 %), including 84 (77 %) with no clinical deterioration and 25 (23 %) with clinical deterioration (Table S1).

### 3.2 Degree of obstruction and clinical outcomes

**Fig. 3:**
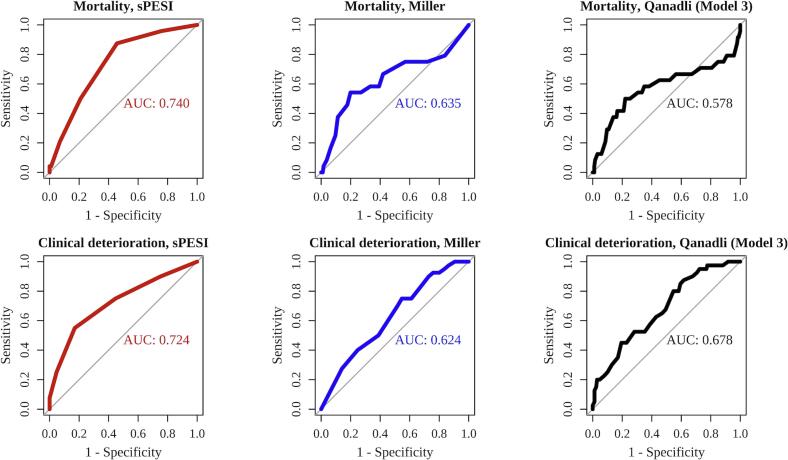
The Area Under the Receiver Operating Characteristic for the sPESI, Miller Index, and the CTOI to Predict Mortality and Clinical Deterioration. Abbreviations: CTOI, computed tomography obstruction index; sPESI, simplified Pulmonary Embolism Severity Index.

The sPESI demonstrated predictive ability for mortality in patients with acute PE, with an AUC of 0.740. However, both the Miller index and the CTOI had limited predictive ability of mortality, with AUC values of 0.635 and 0.578, respectively. Additionally, the sPESI exhibits the best performance in predicting clinical deterioration, with an AUC of 0.724. The Miller index and the CTOI can predict clinical deterioration, with AUC values of 0.624 and 0.678, respectively (Fig. 3). The external validation group demonstrated similar results (Fig. S2). The sPESI demonstrated predictive ability for both mortality and clinical deterioration, while the Miller index and the CTOI have low predictive ability for mortality.

### 3.3 Comparison of the predictive ability of different models

We compared models 1 to 6 to assess the impact of different locations (central versus peripheral) and levels of obstruction (partial versus nearly total) on clinical deterioration. Models 1 and 2 were compared to examine whether adding nearly total occluded central pulmonary arteries will improve the predictive ability. Models 1 and 2 exhibit nearly identical predictive abilities with an AUC of 0.70, along with the same accuracy of 0.82. Furthermore, models 3 and 4 were compared to explore the additive effect of central pulmonary arteries. Model 4 demonstrated similar predictive ability (AUC: 0.69) and accuracy (0.81) compared to model 3 (AUC: 0.68 and accuracy: 0.81). Models 5 and 6 were designed to analyze the predictive ability of obstruction of central pulmonary arteries alone. Model 6 has a better predictive ability (AUC: 0.68) compared to model 5 (AUC: 0.63), while both models have similar accuracy (0.81). Finally, model 7 is the summation of statistically significant variables calculated on univariate analysis. It is composed of the first, third, sixth, and eighth segmental arteries of the right pulmonary artery, the third to ninth segmental arteries of the left pulmonary artery, the middle right pulmonary artery, the main pulmonary artery, and the left proximal to distal pulmonary arteries (Table S2). Model 7 demonstrated the best predictive ability among all the models (AUC: 0.72) (Table 2).

**Table 2:** The ROC Analysis of Different Models with and without sPESI in Predicting Clinical Deterioration.

Models	Peripheral	Central	Partial occlusion	Nearly total occlusion	sPESI	AUC (95 % CI)	Accuracy
Model 1	●			●		0.6996 (0.6148–0.7843)	0.8238
Model 2	●	●		●		0.6996 (0.616–0.7832)	0.8238
Model 3	●		●	●		0.6777 (0.5886–0.7669)	0.8095
Model 4	●	●	●	●		0.6895 (0.6015–0.7774)	0.8143
Model 5		●		●		0.6254 (0.5342–0.7167)	0.8095
Model 6		●	●	●		0.6802 (0.5915–0.769)	0.8095
Model 7	Significant segments on univariate analysis					0.7222 (0.6404–0.804)	0.819
Model 1 + sPESI	●			●	●	0.7753 (0.6914–0.8592)	0.8619
Model 2 + sPESI	●	●		●	●	0.7765 (0.6914–0.8615)	0.8571
Model 3 + sPESI	●		●	●	●	0.7777 (0.6937–0.8617)	0.8619
Model 4 + sPESI	●	●	●	●	●	0.7824 (0.699–0.8657)	0.8667
Model 5 + sPESI		●		●	●	0.7418 (0.6467–0.837)	0.8333
Model 6 + sPESI		●	●	●	●	0.7659 (0.6752–0.8566)	0.8476
Model 7 + sPESI	Significant segments on univariate analysis				●	0.8004 (0.7204–0.8805)	0.8619

Abbreviations: AUC, area under curve; ROC, receiver operating characteristic; sPESI, simplified Pulmonary Embolism Severity Index.

The external validation group analysis revealed similar results. Model 6, which is the summation of partially and totally occluded central pulmonary arteries, exhibited the best predictive ability in the external validation group (Table S3).

### 3.4 Integrated discrimination index (IDI) and net reclassification index (NRI)

The addition of sPESI to models 1 to 7 resulted in an improved predicting value, with an increased mean AUC from 0.68 to 0.77 and increased mean accuracy from 0.82 to 0.86. Model 7 plus sPESI demonstrated the best predictive ability with an AUC of 0.80 (95 % confidence interval [CI]: 0.72–0.88), while model 4 plus sPESI achieved the highest accuracy of 0.87 (Table 2, Table S4). The external validation group analysis revealed similar results, with an increased mean AUC from 0.73 to 0.84 and increased mean accuracy from 0.79 to 0.83 after adding sPESI to the models (Table S3). Statistically significant improvements in predicting clinical deterioration were observed based on IDI, with the IDI ranging from 0.02 to 0.09, when comparing models 1 to 7 added to the sPESI with sPESI alone. The NRI demonstrated an increased predictive ability of models 1–7 with the addition of sPESI (Table 3). The IDI and NRI of the external validation group revealed an improvement in the predicting ability of the models in combination with sPESI compared to sPESI alone (Table S5). Overall, the combination of clinical risk factors and the obstruction severity on CTPA provides a better prediction of clinical deterioration.

**Table 3:** The NRI and IDI Comparing Models Plus sPESI with sPESI.

Models	NRI (95 % CI)	IDI (95 % CI)
Model 1 + sPESI	0.1294 (−0.0100–0.2688)	0.0700 (0.0245–0.1156)
Model 2 + sPESI	0.1235 (−0.0163–0.2634)	0.0691 (0.0238–0.1144)
Model 3 + sPESI	0.1294 (0.0096–0.2492)	0.0643 (0.0252–0.1034)
Model 4 + sPESI	0.1353 (−0.0036–0.2742)	0.0721 (0.0303–0.1138)
Model 5 + sPESI	−0.0397 (−0.1259–0.0465)	0.0283 (0.0017–0.0550)
Model 6 + sPESI	0.0926 (−0.0175–0.2027)	0.0559 (0.0216–0.0903)
Model 7 + sPESI	0.1294 (−0.0100–0.2688)	0.0950 (0.0489–0.1412)

Abbreviations: IDI, integrated discrimination improvement; NRI, net reclassification improvement; sPESI, simplified Pulmonary Embolism Severity Index.

### 3.5 Clinical application

**Fig. 4:**
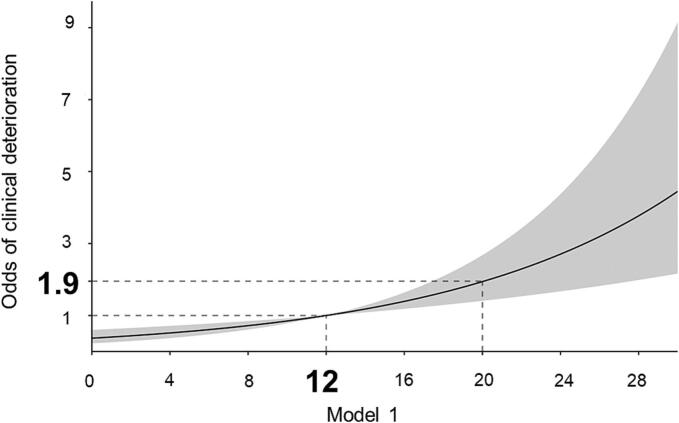
The Odds of Clinical Deterioration Plotted Against Model 1. When the odds ratio is 1, the score of Model 1 was 12.03, while at a score of 20, the odds ratio for clinical deterioration increased to 1.94.

We also plotted the odds of clinical deterioration against Model 1(Fig. 4) and observed that when the odds ratio is 1, the score was 12.03, while at a score of 20, the odds ratio for clinical deterioration increased to 1.94. This suggests that obstruction of fewer than six pulmonary arteries does not significantly increase the likelihood of clinical deterioration. However, occlusion of ten pulmonary arteries was associated with approximately a twofold increase in the risk of clinical worsening. Additionally, nomograms based on the models plus sPESI can be used as a tool in the clinical setting to predict prognosis (Fig. S3).

### 3.6 Inter- and intra-rater variability

Model 3, which is the CTOI, is used to test the intra- and inter-rater variability. 210 cases were used for analysis. The intra- and inter-rater correlation coefficients of model 3 were 0.995 (95 % CI: 0.993–0.996) and 0.989 (95 % CI: 0.986–0.992), respectively (Table S6). The Bland-Altman analysis revealed no intra-rater and inter-rater bias in the calculation of model 3 scores (Fig. S4).

## 4 Discussion

Our study revealed that, in addition to the sPESI, the level of obstruction seen on CTPA also serves as a useful predictor of clinical deterioration. After the addition of central segments and partially occluded segments, the predictivity ability is nearly identical. Therefore, calculating the nearly totally obstructed 20 peripheral segments is an efficient method that balances predictive ability and clinical usability. Combining the severity of obstruction on the image with sPESI improves the predictive ability for clinical deterioration compared to sPESI alone.

Animal studies have revealed that acute pulmonary artery obstruction increases pulmonary artery pressure and pulmonary vascular resistance [15,16]. Severe pulmonary hypertension may cause RV failure, clinical deterioration, and hemodynamic instability. Currently, discrepancies exist in the literature regarding whether PA clot burden could reflect severity. Some studies revealed thrombus burden as a significant predictor of death in patients with acute PE [[17], [18], [19]], but others have not [10,11,14,20]. The ESC recommended against using the anatomical burden and emboli characteristics to determine PE severity [21]. Our study suggests that thrombus burden alone is not the most crucial factor in predicting clinical outcomes. Calculating only the occluded central pulmonary arteries had the lowest predictability of clinical deterioration, possibly because main trunk thrombus does not necessarily reduce distal blood flow. For example, large clots in the main pulmonary artery that only partially obstruct a few pulmonary arteries may not lead to significant clinical deterioration. This finding is contrary to the finding of some previous studies [22,23]. However, based on a recent study, proximal PE was not associated with mortality [24]. When considering clot burden in both the central and peripheral pulmonary arteries, the predictive ability was similar to calculating only the occlusion in the peripheral arteries. Likewise, including partially occluded pulmonary arteries did not improve predictive ability compared to focusing solely on those with nearly total occlusion. This suggests that the key factor in predicting clinical deterioration is the total number of occluded pulmonary arteries rather than the thrombus burden itself. The reduced outflow from these occluded arteries leads to impaired pulmonary circulation, causing RV failure and subsequent clinical deterioration. This explanation may help clarify the controversial results in previous studies on thrombus burden.

The 2019 ESC guidelines on PE management classify patients into high, intermediate, and low-risk groups, and recommended treatment options based on risk strata. However, approximately 5 % of patients initially classified as intermediate risk will develop hemodynamic decompensation [5]. Our study aims to identify methods to predict clinical deterioration in this specific group of patients, where they may benefit from early systemic or catheter-based thrombolytic therapy. Huang et al. used a three-dimensional CT method to estimate the total embolic burden and revealed a positive correlation between the total embolic volume and impending shock [25]. In our study, total obstruction of six peripheral arteries does not increase the odds of clinical deterioration; total obstruction of ten peripheral arteries increases the risk of developing clinical deterioration by nearly two-fold. Additionally, our models demonstrated a positive correlation between the severity of obstruction and clinical deterioration, offering a straightforward and easily applicable method to determine severity in the clinical setting.

The sPESI, which includes age, cancer, chronic cardiopulmonary diseases, heart rate, systolic blood pressure, and oxyhemoglobin levels, can predict the 30-day mortality of patients with PE [13]. Our study revealed that the sPESI could also predict clinical deterioration. However, adding the extent of obstruction of pulmonary improves the ability to predict clinical deterioration since the variables included in the sPESI are not specific to PE alone. Our models increased the predictive ability when added to sPESI, as evidenced by positive NRI and IDI values. The low inter- and intra-observer variability indicates good agreement between the two investigators. The nomogram combining model 1 (totally obstructed peripheral arteries) and the sPESI (Fig. S5) is a simple and reproducible tool to aid physicians in assessing the prognosis of patients with acute PE.

Current treatment guidelines for acute PE recommend systemic thrombolysis for patients who present with hemodynamic instability [4,26], but the management of patients without hemodynamic instability but with unstable clinical features or evidence of RV dysfunction on imaging remains uncertain. Previous studies revealed that treating patients with acute PE with systemic thrombolysis on a background of heparin lowers the risk of developing in-hospital death, clinical deterioration, or hemodynamic decompensation [5,27]. Additionally, a meta-analysis demonstrated that treating acute PE with thrombolytic therapy lowers mortality, but increases the risk of major bleeding [28]. The benefit of thrombolytic therapy may outweigh the risk of bleeding in certain normotensive patients with acute PE with high-risk features. Moreover, due to the emergence of catheter-directed mechanical thrombolysis, due to low risk of bleeding, a more precise prediction method to identify the patients prone to clinical deterioration is essential. In our study, it is demonstrated that in patients who have more than six totally obstructive peripheral pulmonary arteries, the risk of developing clinical deterioration increases. However, clinical decisions based on imaging scores still require large, prospective testing in real-world, diverse populations.

This study has several limitations, including its small sample size and its retrospective design, which prevents assessment of the effects on clinical decision-making and patient outcomes. Additionally, the inclusion of only Asian patients limits the generalizability of the models. Also, given the overlapping AUC and similar accuracies in the Models in the external validation group, it will be our future direction to perform net benefit analysis to provide insights regarding how adding complexity to the models changes the clinical utility.

## 5 Conclusion

In conclusion, our study established a positive correlation between the level of pulmonary artery obstruction and both mortality and clinical deterioration. The combination of calculating nearly totally obstructed peripheral segments and sPESI provides improved predictive ability for clinical deterioration. These findings may offer physicians a tool for predicting the prognosis of patients diagnosed with acute PE.

## CRediT authorship contribution statement

**Jian-Kuan Yeh:** Writing – original draft, Methodology. **Po-Wei Chen:** Writing – review & editing, Validation. **Wei-Ting Chang:** Writing – review & editing, Supervision, Conceptualization. **Pin-Hsuan Chiu:** Formal analysis. **Pei-Fang Su:** Formal analysis. **Chih-Hsin Hsu:** Writing – review & editing, Supervision, Conceptualization. **Chih-Chan Lin:** Writing – review & editing, Project administration, Funding acquisition, Conceptualization. **Hsien-Yuan Chang:** Writing – original draft, Visualization, Validation, Supervision, Resources, Project administration, Methodology, Investigation, Funding acquisition, Data curation, Conceptualization.

## Declaration of competing interest

The authors declare that they have no known competing financial interests or personal relationships that could have appeared to influence the work reported in this paper.
