# Supplementary material for: Simplified computed tomography pulmonary angiography score predicts clinical deterioration in patients with acute pulmonary embolism
Source: Int J Cardiol Heart Vasc. 2025 Jun 3;59:101712. doi: 10.1016/j.ijcha.2025.101712 (PMC12167049; doi:10.1016/j.ijcha.2025.101712)
Supplement: Supplementary Data 1 [file mmc1.docx]

**Supplemental Material**

**Figure S1. Flow Diagram of the Study Cohort**

**
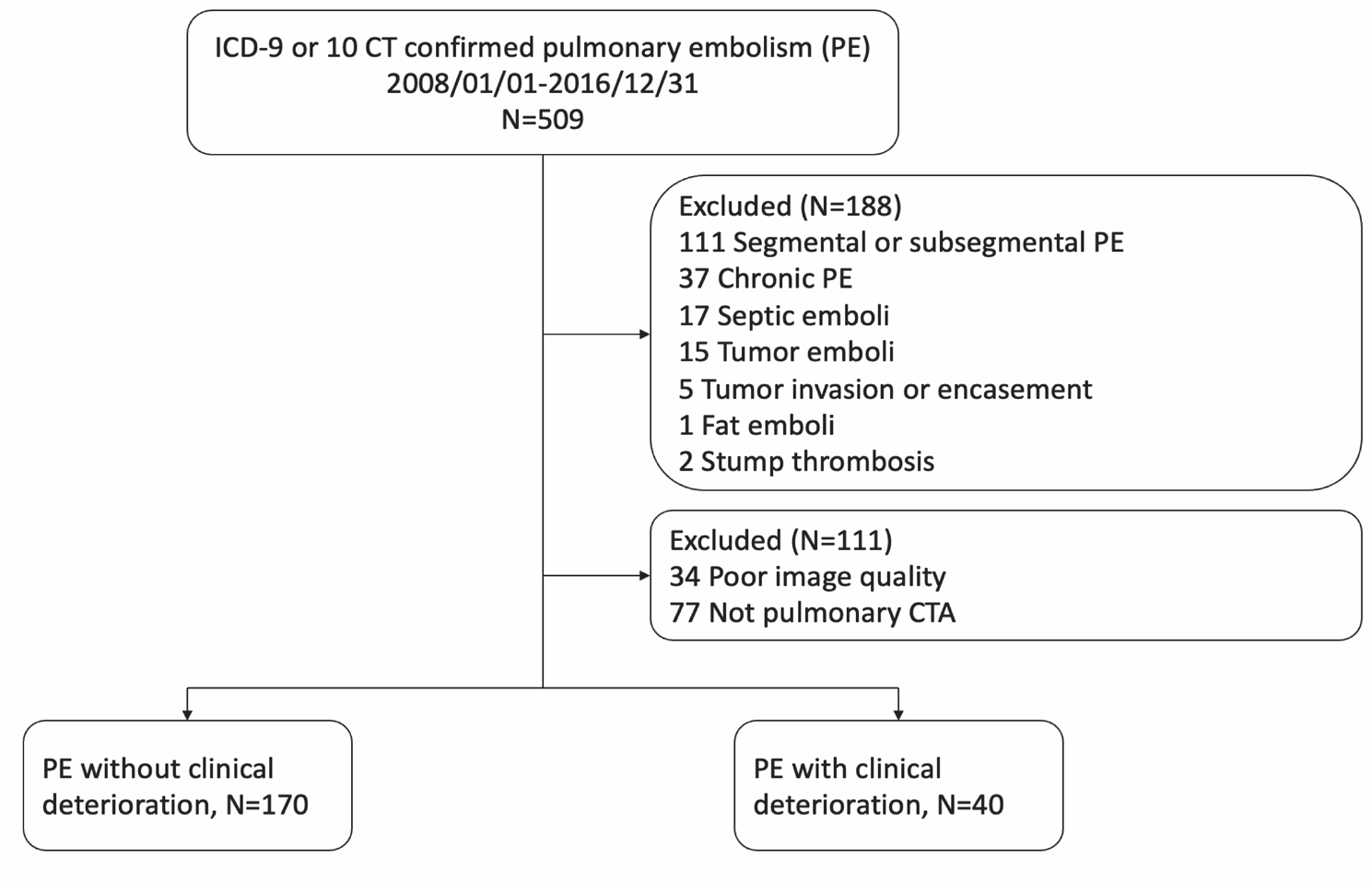
**

Abbreviations: CT, computed tomography; CTA, computed tomography angiography; PE, pulmonary embolism.

**Figure S2. Flow Diagram of the Validation Cohort**

**
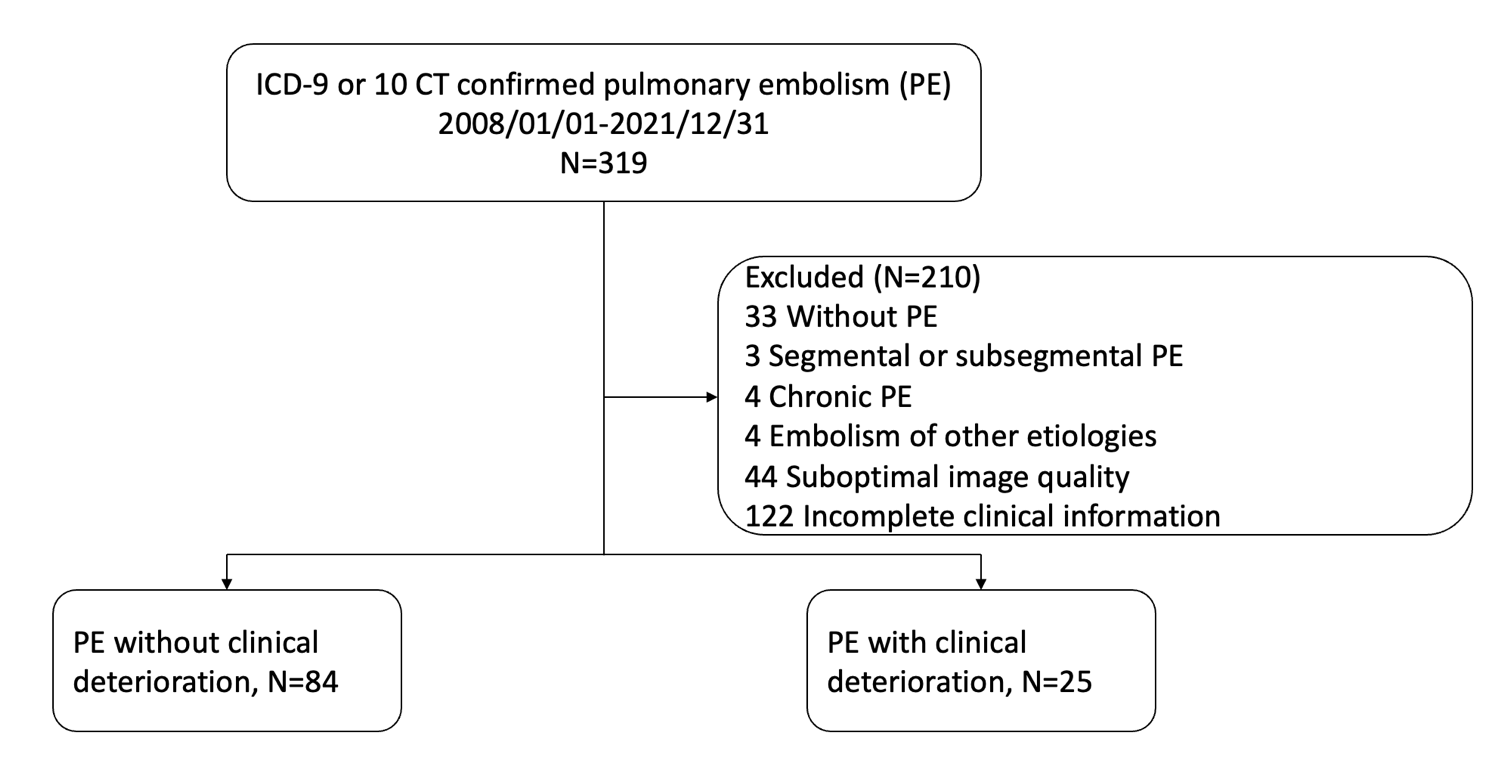
**

Abbreviations: CT, computed tomography; PE, pulmonary embolism.

**Table S1. Baseline Characteristics of Patients with Acute Pulmonary Embolism with and Without Clinical Deterioration in the External Validation Group**

|  | Overall  (N = 109) | With clinical deterioration  (N = 25) | Without clinical deterioration (N = 84) | *P* value |
| --- | --- | --- | --- | --- |
| Age (years old) | 64.61 ± 17.24 | 63.52 ± 14.70 | 64.94 ± 17.99 | .54 |
| Sex (Male) | 47 (43%) | 12 (48%) | 35 (42%) | .65 |
| sPESI | 1.52 ± 1.06 | 2.36 ± 0.95 | 1.27 ± 0.96 | <.01** |
| Cancer | 32 (29%) | 9 (36%) | 23 (27%) | .46 |
| Systemic t-PA | 14 (13%) | 13 (52%) | 1 (1%)*** |  |
| Miller index | 11.05 ± 4.82 | 13.48 ± 3.29 | 10.32 ± 4.98 | <.01 |
| CTOI | 20.85 ± 11.24 | 28.28 ± 8.65 | 18.64 ± 11.01 | <.01 |

Data are presented as mean ± SD or N (%).

**Wilcoxon rank sum test; Fisher's Exact Test for count data

*** Despite receiving systemic tPA, the patient exhibited no clear signs of hemodynamic compromise and was therefore classified in the “non-deterioration” group

Abbreviations: CTOI, computed tomography obstruction index; sPESI, simplified Pulmonary Embolism Severity Index.

**Figure S3. The Area Under the Receive Operating Characteristic for the sPESI, Miller Index, and Qanadli Index**

**
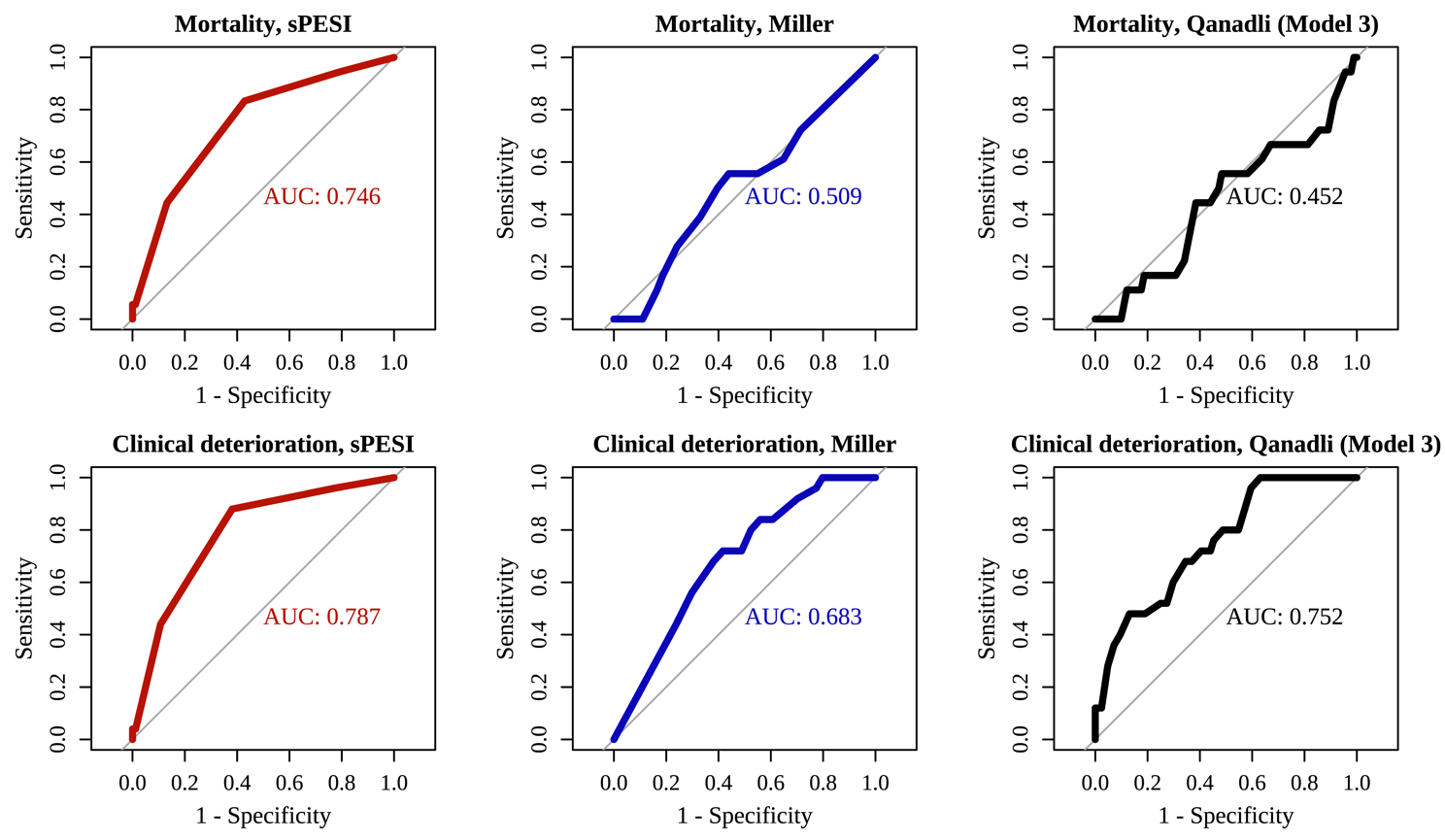
**

Abbreviations: CTOI, computed tomography obstruction index; sPESI, simplified Pulmonary Embolism Severity Index.

**Table S2. Univariate Regression Analysis of sPESI, Different Segments, and Models in Predicting Clinical Deterioration**

| Explanatory | OR (95% CI) | *P* value | Adjust. *P* BH | Adjust. *P* Bonf. |
| --- | --- | --- | --- | --- |
| sPESI | 2.04 (1.53-2.80) | <.001 | <.001 | <.001 |
| RA1 | 1.79 (1.09-3.04) | .026 | .036 | 1.000 |
| RA2 | 1.52 (0.96-2.46) | .078 | .089 | 1.000 |
| RA3 | 2.11 (1.31-3.57) | .003 | .008 | .160 |
| RA4 | 1.50 (0.98-2.36) | .066 | .079 | 1.000 |
| RA5 | 1.20 (0.80-1.83) | .375 | .375 | 1.000 |
| RA6 | 1.58 (1.02-2.49) | .043 | .059 | 1.000 |
| RA7 | 1.56 (1.01-2.48) | .053 | .067 | 1.000 |
| RA8 | 1.97 (1.23-3.31) | .007 | .014 | .336 |
| RA9 | 1.52 (0.96-2.46) | .078 | .089 | 1.000 |
| RA10 | 1.47 (0.93-2.38) | .103 | .112 | 1.000 |
| LA1 | 1.39 (0.89-2.19) | .155 | .158 | 1.000 |
| LA2 | 1.43 (0.93-2.21) | .109 | .116 | 1.000 |
| LA3 | 1.71 (1.11-2.71) | .017 | .026 | .820 |
| LA4 | 1.68 (1.09-2.64) | .021 | .031 | .991 |
| LA5 | 1.76 (1.15-2.76) | .011 | .019 | .546 |
| LA6 | 2.09 (1.35-3.29) | .001 | .003 | .051 |
| LA7 | 1.95 (1.23-3.21,) | .006 | .012 | .287 |
| LA8 | 1.78 (1.13-2.89) | .016 | .026 | .767 |
| LA9 | 1.79 (1.15-2.84) | .011 | .019 | .537 |
| LA10 | 1.52 (0.99-2.38) | .060 | .073 | 1.000 |
| MPA | 2.31 (0.99-5.15) | .045 | .060 | 1.000 |
| RPAp | 1.49 (0.88-2.51) | .134 | .140 | 1.000 |
| RPAm | 2.00 (1.19-3.45) | .010 | .019 | .493 |
| RPAd | 1.58 (0.94-2.70) | .089 | .100 | 1.000 |
| LPAp | 2.76 (1.53-5.10) | .001 | .002 | .0410 |
| LPAm | 2.30 (1.31-4.16) | .001 | .010 | .215 |
| LPAd | 1.74 (1.02-3.04) | .047 | .061 | 1.000 |
| Model 1 | 1.09 (1.05-1.13) | <.001 | <.001 | .002 |
| Model 2 | 1.07 (1.04-1.11) | <.001 | <.001 | .002 |
| Model 3 | 1.09 (1.04-1.14) | <.001 | .001 | .018 |
| Model 4 | 1.08 (1.04-1.12) | <.001 | <.001 | .008 |
| Model 5 | 1.20 (1.05-1.38) | .008 | .015 | .370 |
| Model 6 | 1.27 (1.11-1.46) | .001 | .002 | .032 |
| Model 7 | 1.15 (1.08-1.23) | <.001 | <.001 | .001 |

Abbreviations: Adjust. *P* BH, false discovery rate-adjusted *P* value; Adjust. *P* Bonf., Bonferroni-adjusted *P* value; LA1-10, 1^st^ to 10^th^ segmental pulmonary arteries of the left pulmonary artery; LPAp, proximal left pulmonary artery; LPAm, middle left pulmonary artery; LPAd, distal left pulmonary artery MPA, main pulmonary artery; OR, odds ratio; RA1-10, 1^st^ to 10^th^ segmental pulmonary arteries of the right pulmonary artery; RPAp, proximal right pulmonary artery; RPAm, middle right pulmonary artery; RPAd, distal right pulmonary artery; sPESI, simplified Pulmonary Embolism Severity Index.

**Table S3. The ROC Analysis of Different Models with and without sPESI in Predicting Clinical Deterioration in the External Validation Group**

| Models | Peripheral | Central | Partial occlusion | Nearly total occlusion | sPESI | | AUC (95% CI) | Accuracy |
| --- | --- | --- | --- | --- | --- | --- | --- | --- |
| Model 1 | ● |  |  | ● |  | | 0.7674 (0.6646-0.8701) | 0.7798 |
| Model 2 | ● | ● |  | ● |  | | 0.7650 (0.6627-0.8673) | 0.7706 |
| Model 3 | ● |  | ● | ● |  | | 0.7517 (0.6491-0.8542) | 0.7706 |
| Model 4 | ● | ● | ● | ● |  | | 0.7764 (0.6768-0.8761) | 0.8257 |
| Model 5 |  | ● |  | ● |  | | 0.5600 (0.4551-0.6649) | 0.7706 |
| Model 6 |  | ● | ● | ● |  | | 0.7993 (0.7030-0.8955) | 0.7982 |
| Model 7 | Significant segments on univariate analysis | | | | |  | 0.7660 (0.6677-0.8642) | 0.7890 |
| Model 1 + sPESI | ● |  |  | ● | ● | | 0.8552 (0.7722-0.9383) | 0.8440 |
| Model 2 + sPESI | ● | ● |  | ● | ● | | 0.8538 (0.7724-0.9352) | 0.8440 |
| Model 3 + sPESI | ● |  | ● | ● | ● | | 0.8529 (0.7685-0.9372) | 0.8440 |
| Model 4 + sPESI | ● | ● | ● | ● | ● | | 0.8648 (0.7878-0.9417) | 0.8349 |
| Model 5 + sPESI |  | ● |  | ● | ● | | 0.7910 (0.6948-0.8871) | 0.7798 |
| Model 6 + sPESI |  | ● | ● | ● | ● | | 0.8826 (0.8117-0.9535) | 0.8073 |
| Model 7 + sPESI | Significant segments on univariate analysis | | | | | ● | 0.8583 (0.7794-0.9372) | 0.8349 |

Abbreviations: AUC, area under curve. ROC: receiver operating characteristic; sPESI, simplified Pulmonary Embolism Severity Index.

**Table S4. Multivariate Regression Analysis of Combination of Models Plus sPESI in Predicting Clinical Deterioration**

|  | Model 1 + sPESI | | Model 2 + sPESI | | Model 3 + sPESI | | Model 4 + sPESI | |
| --- | --- | --- | --- | --- | --- | --- | --- | --- |
| Explanatory | OR (95% CI) | *P* value | OR (95% CI) | *P* value | OR (95% CI) | *P* value | OR (95% CI) | *P* value |
| Model 1 | 1.08 (1.03-1.12) | .001 |  |  |  |  |  |  |
| Model 2 |  |  | 1.06 (1.03-1.10) | .001 |  |  |  |  |
| Model 3 |  |  |  |  | 1.08 (1.03-1.14) | .001 |  |  |
| Model 4 |  |  |  |  |  |  | 1.07 (1.03-1.12) | .001 |
| sPESI | 1.96 (1.45-2.73) | <.001 | 1.96 (1.44-2.72) | <.001 | 2.01 (1.49-2.79) | <.001 | 2.00 (1.48-2.78) | <.001 |

|  | Model 5 + sPESI | | Model 6 + sPESI | | Model 7 + sPESI | |
| --- | --- | --- | --- | --- | --- | --- |
| Explanatory | OR (95% CI) | *P* value | OR (95% CI) | *P* value | OR (95% CI) | *P* value |
| Model 5 | 1.18 (1.02-1.36) | .026 |  | . |  |  |
| Model 6 |  |  | 1.25 (1.09-1.44) | .002 |  |  |
| Model 7 |  |  |  |  | 1.14 (1.07-1.23) | <.001 |
| sPESI | 2.01 (1.50-2.76) | <.001 | 2.01 (1.49-2.78) | <.001 | 2.00 (1.47-2.80) | <.001 |

Abbreviations: sPESI, simplified Pulmonary Embolism Severity Index.

**Table S5. The NRI and IDI Comparing the Models Plus sPESI with sPESI in the External Validation Group**

| Models | NRI (95% CI) | IDI (95% CI) |
| --- | --- | --- |
| Model 1 + sPESI | 0.2919 (0.1144-0.4694) | 0.1360 (0.0685-0.2035) |
| Model 2 + sPESI | 0.2919 (0.1144-0.4694) | 0.1060 (0.0501-0.1620) |
| Model 3 + sPESI | 0.2919 (0.1144-0.4694) | 0.1184 (0.0595-0.1773) |
| Model 4 + sPESI | 0.3081 (0.1208-0.4953) | 0.1581 (0.0860-0.2302) |
| Model 5 + sPESI | 0.0119 (-0.0113-0.0351) | -0.0152 (-0.0322-0.0019) |
| Model 6 + sPESI | 0.3005 (0.1056-0.4953) | 0.1826 (0.1054-0.2598) |
| Model 7 + sPESI | 0.3081 (0.1208-0.4953) | 0.1750 (0.0900-0.2599) |

Abbreviations: IDI, integrated discrimination improvement; NRI, net reclassification improvement; sPESI, simplified Pulmonary Embolism Severity Index

**Table S6. The Inter- and Intra-rater Variability**

|  | ICC | 95% CI | *P* value |
| --- | --- | --- | --- |
| Inter-rater reliability | 0.989 | 0.986-0.992 | <.001 |
| Intra-rater reliability | 0.995 | 0.993-0.996 | <.001 |

Abbreviations: ICC, intraclass correlation coefficient.

**Figure S4. The Odds of Clinical Deterioration Plotted Against Model 1**

**

**

**Figure S5. Nomogram Combining Model 1 and sPESI**

**
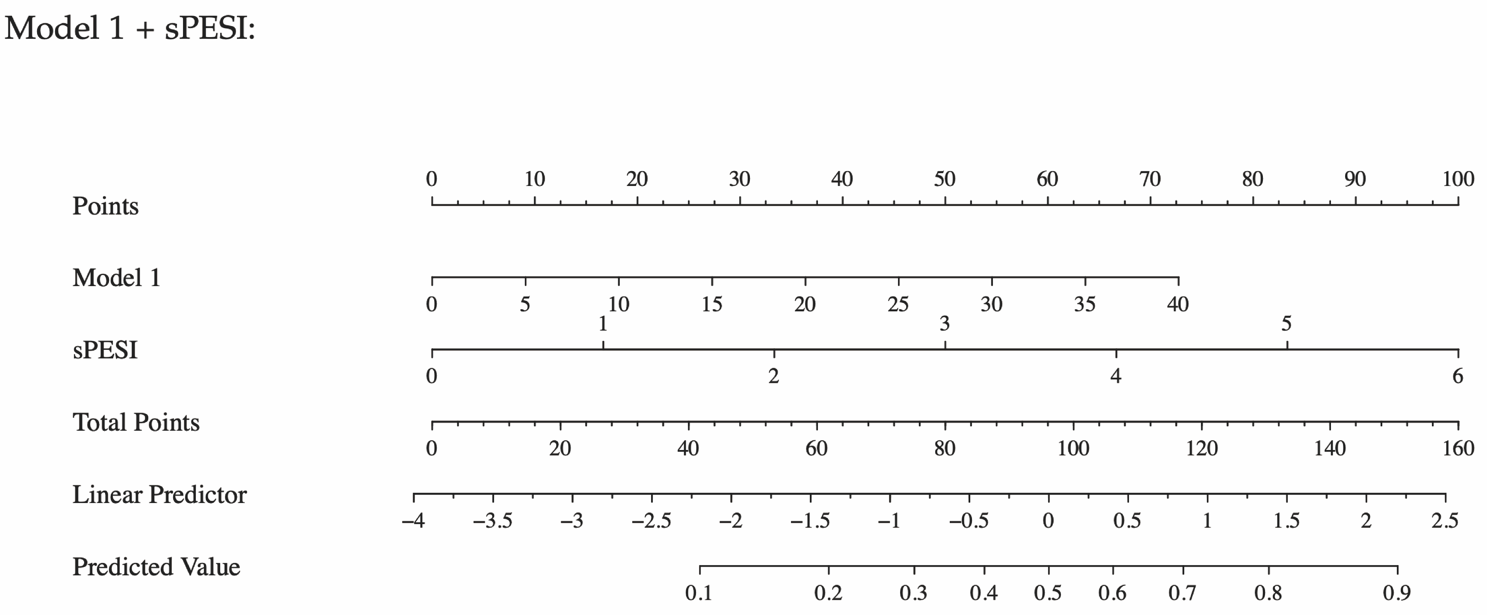
**

Abbreviations: sPESI, simplified Pulmonary Embolism Severity Index.

**Figure S6. The Bland-Altman Plot**

**

**
